# Effect of polyunsaturated fatty acids on secretory phospholipase A2 type IIa in ectopic endometrial cells 

**Published:** 2012-07

**Authors:** Korosh Khanaki, Ali Motavalizadeh Ardekani, Alieh Ghassemzadeh, Vahideh Shahnazi, Mohammad Reza Sadeghi, Masoud Darabi, Amir Mehdizadeh, Abotaleb Saremi, Jafar Soleimani-Rad, Ali Reza Imani, Mohammad Nouri, Ali Rahimipour

**Affiliations:** 1 *Department of Clinical Biochemistry, Faculty of Medicine, Tabriz University of Medical Sciences, Tabriz, Iran.*; 2 *NanoTechnologyResearchCenter, TabrizUniversity of Medical Sciences, Tabriz, Iran.*; 3 *Reproductive Biotechnology Research Center, Avicenna Research Institute, ACECR, Tehran-Iran.*; 4 *Women’s ReproductiveHealthResearchCenter, AlzahraHospital, TabrizUniversity of Medical Sciences, Tabriz, Iran.*; 5 *SaremCellResearchCenter (SCRC), Sarem Women's Hospital, Tehran,Iran.*; 6 *Department of Physiology, Faculty of Medicine, TehranUniversity of Medical Sciences, Tehran, Iran. *; 7 *Faculty of Paramedical Sciences, ShahidBeheshtiUniversity of Medical Sciences, Tehran, Iran.*

**Keywords:** *Fatty acids*, *Endometriosis*, *Secretory phospholipase A2 type IIa*, *Cell culture*

## Abstract

**Background**: Endometriosis is a common chronic inflammation which leads to infertility and chronic pelvic pain in affected women. Secretory phospholipase A2 type IIa (sPLA2IIa) is an acute phase reactant that is markedly increased in inflammatory disorders.

**Objective:** To assess the effects of ω-3 and ω-6 polyunsaturated fatty acids (PUFAs) administration in endometrial cells culture on sPLA2IIa level and cell survival comparing homolog ectopic versus eutopic endometrial cells from endometriosis patients.

**Materials and Methods:** In this experimental study, ectopic and eutopic endometrial tissue samples obtained from 15 endometriosis patients were immediately frozen. After thawing and tissue digestion, mixed stromal and endometrial gland cells were cultured for 8 days in three different culture media; balanced ω-3/ω-6, high ω-3 and high ω-6 PUFAs ratio. Cell survival was measured using 2, 3-bis (2-methoxy-4-nitro-5-sulfophenyl)-5-(phenylamino) carbonyl-2H- tetrazolium hydroxide (XTT) method and sPLA2IIa level assessed with ELISA technique.

**Results:** The sPLA2IIa level was significantly higher in the ectopic endometrial cell culture compared to the eutopic group for each of the three matched treatments (balanced, high ω-3 and high ω-6). Also the sPLA2IIa level in the ectopic endometrial cell group was remarkably increased by each of the three PUFAs treatments compared to control condition (p<0.05, p<0.01, p<0.05 respectively). Cell survival in the eutopic group was significantly decreased by high ω-6 culturing compared to control medium (p<0.05).

**Conclusion:** The increase in sPLA2IIa level in ectopic endometrial cells by fatty acid treatments (especially high ω-3), strengthens the hypothesis that PUFAs stimulate secretion of cytokines leading to increased sPLA2IIa level.

## Introduction

Endometriosis is a frequent gynaecological disorder characterized by the presence of tissue containing functional endometrial glands and stroma outside the uterine cavity (1). The etiology of endometriosis remains incompletely understood, in part, to its multifactorial characteristics (2). 

Endometriosis is associated with a chronic inflammatory response within the peritoneal cavity (3) leading to major problems for women during their reproductive years, such as pelvic pain and infertility (4). Eicosanoids are powerful inflammatory agents that may influence disease-associated pain and infertility (5, 6) and also contribute in the molecular and cellular processes responsible for endometriotic damage (7, 8) such as endometriotic cell survival, invasion (9) and endometrial cell proliferation (10). 

The precursors of prostaglandins, eicosapentaenoic acid (20:5 ω-3) and arachidonic acid (20:4 ω-6) are essential dietary constituents (11). Dietary changes can directly lead to prostaglandin synthesis alteration. ω-3 and ω-6 FAs treatment may reduce endometriosis-related symptoms, selectively modulating biosynthesis and activity of specific prostaglandins involved in pelvic pain (12). 

Proctor (13) demonstrated that addition of vitamins (B1, B6, E), magnesium, and ω-3 Fatty acids (FAs) (fish oil) to diet induced analgesic and anti-inflammatory properties in endometriosis patients. Gazvani *et al* found that high ω-3: ω-6 FA ratio reduced endometrial-cell survival in primary mixed culture of epithelial and stromal cells from endometriosis patients and control subjects (11).

Secretory phospholipase A2 type IIa (sPLA2IIa) is an acute phase reactant markedly increased in inflammatory disorders. sPLA2IIa is a key enzyme in the biosynthesis of eicosanoids by hydrolysing polyunsaturated fatty acids (PUFAs) resulting in the generation of free arachidonic acid and lysophospholipids, precursors of proinflammatory lipid mediators like prostaglandin E_2_ (14). sPLA_2_IIa was the most up-regulated gene in ectopic in comparison with eutopic endometrium (15) and sPLA_2_IIa mRNA was considerably increased in peritoneal lesions compared with matched eutopic endometrium of endometriosis patients (16). 

Also, sPLA2IIa contributes in angiogenesis of endometriosis (17). Fatty acids constitute the initial elements for eicosanoids synthesis. Cellular mediators produced during eicosanoid biosynthesis pathway have a key role in inflammation processes. In this way there is a reciprocal effect between fatty acids and PLA_2_IIa that plays important roles in the regulation of inflammation. 

Assessment of a possible relationship between ω-3 and ω-6 FAs and PLA_2_IIa as an intracellular inflammation signalling molecule could be helpful in exploring the pathogenetic mechanisms and developing medical treatments for endometriosis. The aim of this study was to evaluate the effects of ω-3 and ω-6 PUFAs administration in endometrial cells culture medium on sPLA_2_IIa level and cell survival comparing homolog ectopic versus eutopic endometrial cells from patients with endometriosis.

## Materials and methods

**Patients and sample collection**

Women undergoing laparoscopy (Ackermann instrument GmbH, Germany) for infertility or pain at the Infertility Clinic of Avicenna Centre and Sarem Hospital, Tehran, with endometriosis histologicaly verified, were selected for this study. All patients gave oral consent and the study was approved by the ethics committee of Avicenna research center. 

All participants were infertile with had regular cycles, none had received anti-inflammatory drugs during last three months prior to surgery and all patients were between 18-42 years old. Stage I or II endometriosis was diagnosed according to the revised American Fertility Society (AFS) classification (18). Ectopic endometrial lesions were biopsied by laparoscopy whereas eutopic endometrial sample was obtained by dilatation and curettage from each patient. 

Ectopic tissues were obtained from one of the ovaries or the peritoneum. The phase of menstrual cycle was histologicaly confirmed as secretoric (19). Ectopic and eutopic endometrial tissues were transferred to Dulbecco's Modified Eagle Medium: Nutrient Mixture F-12 (DMEM/F12) phenol red free culture media with final concentration 100 IU/ml penicillin, 100 μg/ml streptomycin (pen-strep) and transported to the laboratory. 

**Preparation of Mixed Stromal and Endometrial Gland Cell Culture**

Because of small sample sizes, samples were immediately frozen (20). Seven tissue samples from stage-I endometriosis and eight from stage-II were prepared. The tissues were simultaneously thawed using 40^o^C water and washed to remove Dimethyl sulphoxide (DMSO). To obtain sufficient cell numbers for experiments, specimens from three or four patients with similar stage of disease were pooled, leading to two Stage I and two Stage II samples of both the ectopic as well as eutopic samples.

The tissues were gently minced and incubated for 90 minutes at 37^o^C in DMEM/F12 containing collagenase D (2 mg/ml) and DNAse I (0.05 mg/ml). After digestion, the suspension was filtered through 100 µm cell strainer to remove debris and undigested material (21). The cell pellet consisting of mostly endometrial gland and stromal cells were resuspended and cell viability was evaluated by trypan blue (11). Evaluation by light microscopy after 24h of culture revealed both tadpole-shaped epithelial and fibroblast-like stromal cells in the culture (22). Epithelial cells tended to cluster like glands in contrast to stromal cells which were predominantly single cells. 

Ectopic and eutopic endometrial cells were plated in 96 well culture dishes (BioHit, Canada) at a density of 50000 and 10000 for ELISA and 2,3-bis (2-methoxy-4-nitro-5-sulfophenyl)-5-[(phenylamino) carbonyl] -2H-tetrazolium hydroxide (XTT) proliferation (survival) assays respectively. DMEM/F12 was supplemented with 1.2 mg/ml NaHCO_3_, 100 IU/ml penicillin-100μg/ml streptomycin, 50 μg/ml gentamycin, 5% fetal bovine serum (FBS) and Insulin- Transferrin-Selenite (ITS) [(containing insulin (10 µg/ml ), transferrin (5.5 µg/ml) and selenite (5 ng/ml)]. For cell attachment, the medium was changed after 48 hours and replaced with DMEM/F12 with 0.1% bovine serum albumin (BSA) for 12 hours. 

The PUFAs were added to the modified starved medium (Table I), in which BSA was replaced with 0.7% free fatty acid bovine serum albumin (Free FA BSA). The concentrations of PUFAs were selected according to their physiological levels in human plasma (11). The cells were cultured for more eight days in their respective media as follows with renewal of the media after four days.

**Experimental protocols**

Effect of treatment with PUFAs on ectopic and eutopic samples were separately evaluated as below: 1) Control group: medium without PUFAs. 2) Balanced PUFAs group: medium with balanced ω-3: ω-6 PUFAs ratio, 3) High ω-3: ω-6 PUFAs ratio group: medium with high amounts of ω-3 PUFAs and 4) High ω-6: ω-3 PUFA ratio group: medium with high amounts of ω-6 PUFAs. The relative PUFA composition and concentration is presented in table I. 

**XTT proliferation (survival) assay**

XTT, a yellow tetrazolium salt, is cleaved to a soluble orange formazan only by metabolically active cells, these assays detect viable cells exclusively, Since proliferating cells are metabolically more active than non-proliferating (resting) cells, the assays are suitable not only for the determination of cell viability but also for the determination of cell activation and proliferation.

Cell survival activity was assessed eight days after initiation of PUFAs treatments by using XTT (23). The results of each of the three PUFAs treatments were normalized with the control medium and relative XTT activity was calculated as a percentage of the control (activity of control was considered as 100). The experiments were done as triplicate. 

**Measurement of sPLA**_2_ **IIa**

Prior to treatment and eight days after treatment, the cultured cells were washed twice with ice-cold PBS and lysed with sandwich ELISA lysis buffer (Cell Signalling) without ethylene glycol tetra acetic acid (EGTA). The total protein content in lysates was measured using the bicinochoninic acid microplate system (BCA Protein Assay Kit, Pierce). sPLA_2_IIa assay was performed using enzyme immuno assay (EIA) kits (Cayman Chemical) and ELISA reader (Bio-Tek, Canada). The detection limit for sPLA_2_IIa was 15.6 pg/ml.

**Materials**

DMEM/F12, penicillin-streptomycin, DMSO, trypan blue, gentamycin, ITS, BSA, Free FA BSA, Tris, FAs and XTT were obtained from Sigma-Aldrich Co. cell strainer was obtained from BD Falcon Co. FBS was acquired from Gibco Co and collagenase D and DNAse I were obtained from Roche Co. 

**Statistical analysis**

Data are expressed as means±SEM. To compare sPLA_2_IIa level and XTT activity between and within ectopic and eutopic groups, the Kruskal-Wallis and Mann-Whitney tests were used. Significant differences were presented as p<0.05.

**Table I:** Composition of polyunsaturated fatty acid treatment culture media

**Fatty acid in the cultured medium**	**Control** **(no PUFA)**	**Balanced ω3:ω6** **PUFA ratio**	**High ω3:ω6** **PUFA ratio**	**High ω6:ω3** **PUFA ratio**
Palmitic acid a	0	20.8	20.8	20.8
Oleic (ω-9) acid a	0	20.4	20.4	20.4
Linoleic acid (ω-6) a	0	8	8	8
α-Linolenic acid (ω-3) a	0	0.4	0.4	0.4
Arachidonic acid (ω-6) a	0	2.4	-	4.8
Eicosapentaenoic acid (ω-3) a	0	2.4	4.8	-
Fatty acid free bovine serum albumin in Tris 0.1M PH=8	0.7%	0.7%	0.7%	0.7%

PUFA = Polyunsaturated fatty acid.

a =Mass of fatty acid (µg) per mL DMEM/F12.

## Results

**Figure 1:**
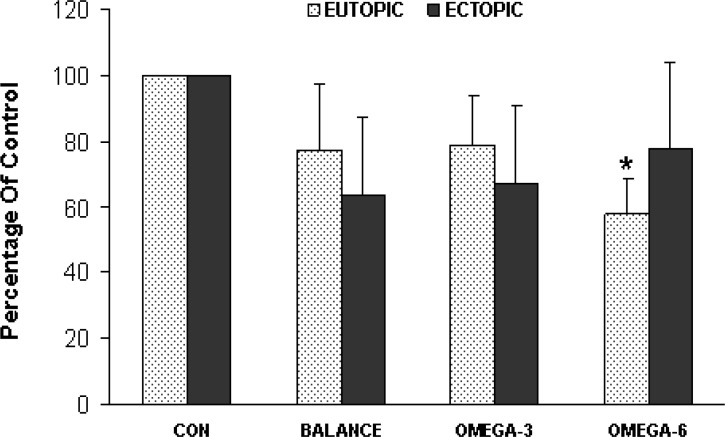
Proliferation (survival) activity in the two cell groups (ectopic and eutopic endometrial cells) from the endometriosis patients under matched culture treatments

Proliferation (survival) activity did not differ significantly between the two groups (of ectopic and eutopic endometrial cells) from the endometriosis patients under any of the matched culture treatments. However within the eutopic group, cell survival significantly decreased during high ω-6 intervention compared with control medium (Figure 1). 

The level of sPLA_2_IIa in the ectopic endometrial cells group was higher (although not statistical significant; 2.172±0.707 vs. 1.282±0.448 pg/µg total protein; p=0.2) than the eutopic group when measured under pre-treatment condition. Because of the large 

subject to subject variation in sPLA_2_IIa level and changes in total cell proteins, results were normalized as level per total cell protein and expressed as percentages of sPLA_2_IIa level under control conditions on day eight after treatment for further analysis. 

**Figure 2:**
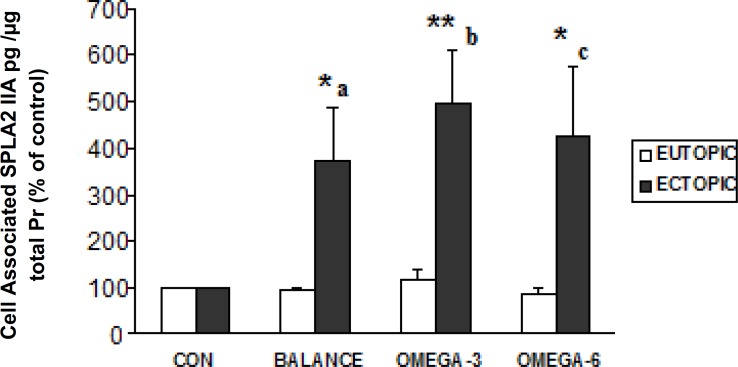
The sPLA_2_IIa level in the two cells groups (ectopic and eutopic endometrial cells) from the endometriosis patients under matched culture treatments. CON: control, BALANCE: balanced ω3: ω6, OMEGA-3: High ω3: ω6, OMEGA-6: High ω6: ω3 .CON: control, BALANCE: balanced ω_3_: ω_6_, OMEGA-3: high ω_3_: ω_6, _OMEGA-6: high ω_6_: ω_3._

Figure 2 summarizes the sPLA_2_IIa level in different groups. sPLA_2_IIa level remarkably increased during each of three PUFAs treatments (balanced, high ω-3 and high ω- 6) within ectopic group compared with control condition (p<0.05, p<0.01, p<0.05 respectively). Also there was significantly enhanced level of sPLA_2_IIa in ectopic compared with eutopic group between each of the three matched PUFAs exposures.

CON: control, BALANCE: balanced ω3: ω6, OMEGA-3: High ω3: ω6, OMEGA-6: High ω6: ω3.

Data has been shown as Mean± SEM. * p<0.05 compared with control within group.

## Discussion

The present study indicates that each of the three PUFAs exposures lead to increase in sPLA_2_IIa level within ectopic endometrial cell group compared with the cells cultured in control media. Also the level of sPLA_2_IIa for each of three PUFAs exposures was significantly higher in ectopic compared with matched eutopic group. Only high ω-6 treatment in eutopic group significantly lowered cell survival compared with control medium. 

Primary mixed stromal and epithelial cell culture is one of the models for in vitro study of endometrium (24). In our study a similar method has been used to assess ectopic and eutopic endometrium. This type of model has been demonstrated to be valuable in reproducing cell behaviour in situ and a physiological basis for investigating molecular and genetic processes of disease (25). Also, cell interaction and paracrine regulation are conserved in this model (26). One of the major hypotheses of endometriosis pathogenesis is that retrograde menstruation leads to the entry of endometrial cells outside the uterus (27). 

According to this a primary combined stromal and epithelial cell culture would be a better model than studying stromal or epithelial endometrial cells separately. In our study, different cell types were present in the retrieved tissue biopsies, and cells cultured from ectopic tissue included glands and stroma, resident macrophages and vascular elements. Therefore, in our model, it is not possible to differentiate between macrophages, epithelial, stromal or vascular cells as the originator of the increased sPLA_2_IIa level. The balanced ω-3: ω-6 PUFA ratio was used to imitate partial composition of principal fatty acid types in plasma triglyceride (28, 29). 

Palmitic acid and oleic acid were utilized as indexes for the total saturated FAs and the total monounsaturated FAs respectively. Relative concentrations of the used essential FAs linoleic acid and α-linolenic acid are similar to plasma levels. Eicosapentaenoic acid and arachidonic acid were utilized as two major long-chain PUFAs for ω-3 PUFA and ω-6 PUFA respectively in line with Gazvani *et al* (11). They demonstrated that exposure to high ω-3 PUFA significantly declined the survival of endometrial cells from endometriosis patients compared with those from women without endometriosis. 

In our study, potential confounders such as menstrual cycle day, hormonal changes, genetic background and nutrition were eliminated since evaluations were done between eutopic and ectopic samples from the same endometriosis women. In this setting it was the high ω-6 PUFA that reduced eutopic endometrial cell survival. 

sPLA_2_IIa level was higher (although not statistically significant) in ectopic compared to eutopic endometrial cells prior to fatty acid treatment. This is in line with PLA_2_ activity being elevated in peritoneal fluid from endometriosis patients (30), the sPLA_2_IIa gene was significantly up-regulated in ectopic versus eutopic endometrium (15) and sPLA_2_IIa mRNA was dramatically increased in peritoneal lesions versus matched eutopic endometrium from endometriosis patients (16). 

sPLA_2_ does not only lead to release of arachidonic acid as originator for prostanoids biosynthesis (14), it may also stimulate inflammatory cells via processes isolated from its enzymatic action (31) and play a role in angiogenesis of endometriosis by induction of vascular endothelial cell migration (17). Since PLA_2_IIa is able to enhance its own expression (32), small increments of PLA_2_IIa could lead to fold enhanced expression as reported in earlier studies (15, 16).

PUFAs and especially high ω-3: ω-6 remarkably enhanced the production of sPLA_2_IIa level in ectopic endometrial cells sampled from our endometriosis patients. Despite of different sampling design, the results are similar to the Gazvani *et al* study (11) that showed endometrial cells from women with endometriosis in comparison to those from women without endometriosis secreted higher concentrations of IL-8 (a proinflammatory and angiogenic cytokine), especially in the presence of high ω-3PUFA ratios. The causes of these results are not clear, the following theories have been proposed: 1) ω- 6 and especially high ω-3 PUFA ratios exposure may induce production of certain cytokines or growth factors (11) leading to increased sPLA_2_IIa level. 2) ω- 6 and especially high ω-3 PUFA may also have effects on other mechanisms that regulate the sPLA_2_IIa level, PLA2 enzyme acts as a repair enzyme for membrane phospholipids during oxidative damage (33, 34). 

Shanti *et al* (35) reported that lipid peroxidation markers were elevated in peritoneal fluid from endometriosis patients and oxidative stress has been suggested as potential factor in initiation and development of endometriotic damage (35, 36). In addition, sPLA2-IIA has anti-tumorigenic property (37). Therefore, the maintenance of sPLA2 as a repair enzyme is possibly advantageous. In line with beneficial effects of ω-3 PUFA that has previously been proved, this may also be linked to the fatty acids effect on sPLA_2_IIa level. 

Although PLA_2_ activity acts as initiator of biosynthesis of prostaglandins and leukotrienes, in order not to deter the useful effects of PLA_2_, application of a selective inhibitor of the inflammatory metabolites is still required (16). In line with Calder PC (38) and several in vivo studies regarding high ω-3 PUFAs intake as potentially effective against inflammation in endometriosis (39) we also regard ω-3 PUFA as an adjuvant in the treatment of endometriosis by reducing the inflammatory reaction and modulating cytokine function and prostaglandin production**. **

## Conclusion

We found that ω-3 and ω- 6 PUFAs, especially high ω-3: ω-6 PUFA ratios increased sPLA_2_IIa level in ectopic endometrial cells sampled from endometriosis patients in culture medium. Further studies are needed to explore the mechanism of PUFAs actions on sPLA_2_IIa concentration in ectopic endometrial cells and also to elucidate different roles of this enzyme in the pathogenesis and treatment of endometriosis.
